# Differences of Host Country-Destination Image Assessment for International Students According to Risk Perception in COVID-19 Tourism

**DOI:** 10.1177/21582440231181592

**Published:** 2023-07-08

**Authors:** NaHyun Lee, Bong-Seok Kim

**Affiliations:** 1 Department of Hospitality Management, College of Hotel & Tourism Management, Kyung Hee University, Seoul, Korea

**Keywords:** destination image, COVID-19, risk perception, revised IPA, international students

## Abstract

The study examined the comparative assessment of destination image according to the perception of COVID-19 and travel risk among international students. The online survey was administered to 786 international students enrolled in universities in Korea. Cluster analysis was performed, and three distinct clusters were identified based on risk perception. Destination image attributes were generated into four underlying dimensions: social environment, tourist environment, destination environment, and imagery, using the exploratory factor analysis. A revised Importance-Performance Analysis (IPA) method was utilized to assess the destination image of Korea and used to compare the expected performance of the attributes on each cluster. In addition, a revised IPA grid of each cluster was presented to unveil the satisfaction attributes of the destination image and suggest communication strategies. This study provides practical insights to destination marketers and organizations to design marketing strategies for international students. Further practical and theoretical implications are discussed.

## Introduction

The outbreak of the COVID-19 pandemic has resulted in an unprecedented impact on the global tourism industry (Gurung, 2021). The travel restriction has unexpectedly affected the flow of travellers worldwide (Yu et al., 2021). Tourism has been one of the areas most affected by the COVID-19 outbreak, and fear of COVID-19 has put destinations on lockdown in many countries (Neuburger & Egger, 2021). Health-related risks have negatively impacted the tourism industry, as travel can play a crucial role in spreading pandemics between destinations (Sánchez-Cañizares et al., 2021). Risk has been considered a primary concern for travellers (Kozak et al., 2007) because an individual inherently pursues the need for safety and security (Beirman, 2002). As the perception of pandemic-related risk may differ among tourists, it is important in the decision-making process when selecting destinations (Bhati et al., 2021) and their future travel decisions (Yüksel & Akgül, 2007). During the COVID-19 pandemic, destination image can be significantly influenced by tourist perceptions of safety and risk (Casali et al., 2021). Moreover, the destination image shaped by media during the COVID-19 outbreak affects willingness to support and travel intention (Rasoolimanesh et al., 2021). Although both destination image and travel risk perceptions have been studied extensively in the past few decades, limited research has been conducted to simultaneously investigate the roles of these two variables (Chew & Jahari, 2014). Addressing this potential research gap, this research examined the risk perception of COVID-19 tourism and destination image perceptions among international students in Korea.

International students are often considered long-term tourists with significant potential to influence their host destination and attract future visitors (Brown, 2009). Although their primary objective is to pursue their studies, they engage in various activities and travel around a host country to promote cultural understanding and have multiple opportunities for interactions (Jamaludin et al., 2016). International students make a number of local trips themselves and strongly influence visiting their family, friends, and relatives during their stay at a host destination (Shani & Uriely, 2012). Prior research on expats in Korea suggests that expats’ assessment of destination image could provide meaningful insights to rescue tourism destinations during the COVID-19 crisis because expats can be considered long-term tourists whose information about destination is more reliable than occasional tourists (Zaman et al., 2021). However, the research lacked links between the perception of risk and destination image. Risk perception can be described as subjective and a central element of destination choice (Bhati et al., 2021; Moreira, 2008); therefore, destination image, according to the risk perception, should be examined. In this regard, this study investigated international students to assess destination image and risk perception while comparing cluster markets characterized differently in their expected performance of the host country’s destination image attributes. To that end, this study adopted an Importance-Performance Analysis, which helps us identify critical attributes and suggests strategies to improve destination image to attract a target group of people (Wu & Jimura, 2019). The results also provided insights into tourism researchers’ and destination marketers’ relationship between destination and risk perception during the COVID-19 pandemic. The objectives of this study are twofold: (1) to evaluate the perceived importance of the attributes forming the destination image based on the perception of COVID-19 and travel risk and; (2) to compare the expected performance of the host country’s destination image attributes in attracting international students in Korea.

## Theoretical Background

### Risk Perception in COVID-19 Tourism

Tourism researchers have researched perceived risk and its impact on travel decision-making and tourist behavior (C. Huang et al., 2020). As health-related diseases such as SARS and MERS have severely impacted the tourism industry, many studies on risk, security, and various aspects of the economic impact of epidemics on travel intention have been released (C.-K. Lee et al., 2012). The term risk has been widely used in tourism research since the 9/11 terrorist attacks in 2001(Williams & Baláž, 2015). Much of the existing literature has supported this concept through research findings showing that tourists tend to avoid destinations with higher potential epidemic risks (Batra, 2008). Risk perception in the tourism industry is defined as an individual’s perception of the likelihood of exposure to risks that may affect travel decisions if the risk is considered to exceed acceptance levels (Chew & Jahari, 2014). As risk perception is very subjective (Dickson & Dolnicar, 2004), it is also described as evaluating the risk of threat situations based on its characteristics and severity (Moreira, 2008). An individual’s subjective assessment of actual risk differs from absolute risk or real risk because people can recognize different levels of risk associated with the same outcome (Fuchs & Reichel, 2011). Individual perception of risk is a significant determinant of human behavior, and many scholars investigated the notion of perceived risk instead of focusing on real risk (Dillard et al., 2012). For instance, the risk perception of travellers predicts the information-seeking process of tourists, which helps them accumulate risk information that influences their travel intentions (Meng et al., 2021). In the present analysis, risk perception is defined as the degree of potential loss an individual perceives, originating from the adverse results of traveling due to the global pandemic caused by COVID-19.

Indeed, risk perception is a central element in the decision-making process when travellers select destinations (Bhati et al., 2021). Studies by Quintal et al. (2010) and Rather (2021) highlighted that the higher the perceived risk from COVID-19, the more negative the attitude to travel during the pandemic. Furthermore, the perception of health risks is negatively related to the perception of safety at a destination (Bae & Chang, 2021) and can influence travelers’ travel intentions (Neuburger & Egger, 2021). In specific, the COVID-19 pandemic on tourist risk perception affects the way tourist visits on destination; for instance, the perception of risk was more significant the longer the period between booking and traveling, as well as the more significant the distance time in traveling to enjoy the trip to the destination (da Silva Lopes et al., 2021). Nazneen et al. (2021) used protection motivation theory to investigate relationships between perceived COVID-19 impacts and travel avoidance. The study demonstrated that travelers’ perceived COVID-19 impacts positively correlated with their travel risk perception, health and safety perceptions, and travel avoidance. Perceived COVID-19 and travel risk perception specify the adverse effects of the pandemic increased the travelers’ fear of traveling. Likewise, the prior research confirms the theoretical link between perceived risk and traveling intention: the riskier a destination is perceived to be about COVID-19, the less likely tourists visit it (Teeroovengadum et al., 2021).

### Host Country-Destination Image and Satisfaction of International Students

#### Destination Image for International Students

Destination image is described as perceptions, impressions, or mental representations that potential visitors have of a place as a travel destination (Hahm et al., 2018). Destination image comprises the sum of cognitive opinions and affective impressions tourists collect and remember about a specific place (Akgün et al., 2020). The cognitive component designates the sum of beliefs or knowledge about the attributes of a specific destination (Pike & Ryan, 2004), while the affective component refers to the psychological impressions people have of a destination (Byon & Zhang, 2010). Thus, the destination image is the collective perception of the attributes or attractions available within a destination (Hallmann et al., 2015). Previous tourism research has found that destination image has a significant direct effect on positive tourist behaviors (Qu et al., 2011), such as revisit intentions, willingness to recommend, intention to visit, the usage of giving word of mouth (Prayag & Ryan, 2012; Tasci & Gartner, 2007). Since tourists tend to choose a destination with a positive image (Leisen, 2001), image is important for understanding and measuring future tourism development. International students are often considered long-term tourists with significant potential in the tourism industry since they shape a substantial market as an economic growth engine (Jamaludin et al., 2016). The main objective is to pursue their studies, yet, they often travel to the host country to deepen their socio-cultural understanding and promote adaptation to the new culture (N. Lee & Kim, 2021; Min-En, 2006). The tourism experience of international students naturally creates the image of the host country’s destination, which causes the formation of Korea’s national image and tourism behavior (U.-K. Lee, 2021). Furthermore, Brown (2009) highlighted their influence on attracting future visitors, such as family or friends, to their host destination during their study (Shani & Uriely, 2012). The image of a country or region as a travel destination has been examined to understand their perception of the host country-destination attractiveness and travel behaviors (Hsiao et al., 2016). Considering international students’ importance in the tourism industry, thorough attention from tourism researchers is required (Bae & Song, 2017).

#### Destination Image and Tourist Satisfaction

In tourism studies, the concept of satisfaction is referred to as the pleasure that the tourist feels due to the ability of the tourist experience to meet their expectations and demands in terms of tourist experiences (Chen & Tsai, 2007). Satisfaction is shaped by subjective comparisons between perceptions and customer expectations (Ozdemir et al., 2012) and comprises affective and cognitive aspects that are primarily decided during the visit (De Rojas & Camarero, 2008). Therefore, the destination image is considered a direct antecedent of satisfaction in tourism research (Chen & Phou, 2013; B. Lee et al., 2014). Numerous studies establish the effects of destination image on tourist satisfaction (Bigne et al., 2001) and support that it is an important component in tourist satisfaction. For example, tourists’ positive image of a destination affects their satisfaction (Hasan et al., 2020), and these findings are also in line with previous studies conducted by Chi and Qu (2008), who believe that the more favorable a destination’s image is, the better the satisfaction. In this study, satisfaction is defined as tourists’ evaluation of the destination compared to their cognitive and affective image expectations.

## Importance-Performance Analysis and Tourism Research

### Revised Importance-Performance Analysis

The importance-performance analysis (IPA) proposed by Martilla and James (1977) is a widely applied simple analysis method to understand the perceptions of customers about the main products or service attributes (Caber et al., 2012). Researchers mostly prefer this analysis technique because it is easy to estimate corporate competitiveness in the market, identify opportunities for improvement, and guide strategic marketing decisions (Martilla & James, 1977; Myers, 1999). Based on the four-quadrant graph consisting of the importance attribute described as the *x*-axis and the performance attribute described as the *y*-axis, researchers can indicate the significant weaknesses and provide a way to enhance customer satisfaction (Martilla & James, 1977; Matzler et al., 2004). However, the traditional IPA technique has shortcomings (S. Huang, 2010). Several researchers have attempted to address the shortcomings of the IPA model by providing revised versions. For example, Matzler et al. (2003) suggested that the implicit importance could be calculated using the partial correlation with the overall satisfaction to eliminate co-linearity. Deng (2007) extended the potential by providing that the link between the attributes and the overall satisfaction was not necessarily linear, as attributes had to be converted to natural logarithms first. Furthermore, Deng (2007) suggested that the conversion approach can address a possible issue with a linear and symmetrical link between attribute performance and overall customer satisfaction. In addition, the potential problem of multicollinearity among independent variables can also be eliminated since it utilizes partial correlation analysis (Kim et al., 2014).

### IPA Approach to Destination Image

Using the IPA method allows tourism stakeholders to diagnose underlying deficiencies and set priorities in tourism development for tourism destinations (Sever, 2015). The studies applying IPA are often presented at destination issues utilized to measure the service quality, image, and overall destination performance (Murdy & Pike, 2012). Thus, a more efficient allocation of limited destination attributes could enhance tourist satisfaction and competitiveness (Jeng et al., 2019). In order to investigate destination image utilizing an IPA method, insight from prominent and widely recognized conceptual destination image studies helps us identify critical attributes. The method proposes ways to promote destination image in order to attract a certain group (Wu & Jimura, 2019). Several researchers investigated the concept of destination image utilizing the IPA method in various tourism studies. For instance, Huang et al. (2022) used the IPA matrix to visually reveal the distinct differences between the projected image by Destination Management Organizations (DMOs) and the perceived image of tourists and proposed strategies that DMOs should adopt at various dimensions. Another image study was conducted by Caber et al. (2012), who employed IPA for the general destination attributes to identify the perception differences toward these attributes among four segmented groups. Additional research on destination images utilizing IPA was used in prior studies (Lin & McDowall, 2012; Liu, 2010).

## Methodology

### Research Design

An empirical study case is presented to illustrate the application of the revised IPA for international student satisfaction of destination image in COVID-19 tourism. The constructs included risk perception and measurement of destination image followed by sociodemographic questions. The construct of risk perception was operationalized with eight items suggested by previous studies (Cahyanto et al., 2016; Neuburger & Egger, 2021; Quintal et al., 2010; Rather, 2021). Participants were asked to measure each item on a 5-point Likert scale, ranging from extremely disagree to extremely agree. The construction of the destination image measurement scale used 17 cognitive and affective images from previous studies (Akgün et al., 2020; De la Hoz-Correa & Muñoz-Leiva, 2019; Xu et al., 2018). In order to apply the revised IPA, this study does not use expectations to measure customer satisfaction (Deng, 2007; Kim et al., 2014). All questions of destination image attributes were rated using a 5-point Likert scale, ranging from not at all satisfied to extremely satisfied. In addition, one question for overall satisfaction was scored on a 5-point Likert’s scale. The questionnaire was prepared in two versions: Korean and English. Before the formal survey, a pilot study was performed for 50 international students to modify any misleading or ambiguous items.

### Data Collection and Sample Characteristics

An online survey was used to effectively reach a broader range of respondents across different regions in Korea. The questionnaire was electronically sent to 1,500 international students randomly selected from the international student community in Korea. The data were collected for 2 weeks, from 11 to 25 February 2022. A total of 790 completed questionnaires were collected, and four out of them were excluded from the data analysis since they chose the same score for all Likert’s scale questions. After excluding unusable responses, a total of 786 usable responses were retained for analysis. The characteristics of the survey respondents are shown in Table 1.

**Table 1.** Characteristics of the Survey Respondents (*n* = 786).

Characteristics	*n*	%	Characteristics	*n*	%
Gender			Age (years)		
Male	361	45.9	18–24	200	25.4
Female	425	54.1	25–30	428	54.5
Nationality			31–36	123	15.6
Africa	111	14.1	Over 37	35	4.5
Northeast Asia	41	5.2	Length of residence (years)		
Southeast Asia	319	40.6	0–<2	194	24.7
Central Asia	93	11.8	2–<4	451	57.4
Europe	65	8.3	4–<6	125	15.9
America	99	12.6	6+	16	2.0
Middle East	58	7.4	Test of proficiency in Korean (TOPIK)		
Program of study					
BA	194	24.7	Level 1–3	190	24.2
MA	443	56.4	Level 4–5	415	52.8
Doctoral	149	19.0	Level 6	181	23.0

## Results

### Quantitative Procedures to Derive Factors

#### Perception of COVID-19 and Travel Risk

Exploratory factor analysis was employed using principal component analysis with a varimax rotation to verify the construct validity and Cronbach’s α value for each dimension for reliability. The Kaise-Meyer-Olkin (KMO) overall measure of sampling adequacy was 0.826, and Bartlett’s sphericity test was significant (χ^2^ = 3,272.119, *p* < 0.001). The EFA results indicated Eigen-values greater than 1.0, factor loadings greater than 0.40, and a total variance indicated 70.496%. The eight risk perception statements were classified into two dimensions: perception of COVID-19 and travel risk perception. Cronbach’s α coefficients of each dimension showed .860 and .881, respectively. The results showed that the construct validity of the questionnaire was acceptably valid, and the questionnaire scales have considerable reliability.

#### Three Clusters of Risk Perception in COVID-19 Tourism

Based on risk perception in COVID-19 tourism, the three distant clusters were defined by performing cluster analysis. First, this hierarchical clustering analysis method was utilized to apply the stopping rule to determine the number of clusters where the homogeneity of clusters is increasing relatively significantly. Then, the *k*-means method was conducted, and clusters were divided into three based on COVID-19 and travel risk perception. Significant differences were found in the analysis of variance between all clusters regarding the construct perception of COVID-19 and travel risk perception. Cluster 1 “The Anxious,” comprises 49% of the sample. The results showed the highest perception of COVID-19 and travel risk perception among the clusters. Cluster 2 “The Reserved,” accounts for 29% of participants. The cluster perceives the perception of COVID-19 as lower means in all three constructs than the other clusters; however, the cluster showed a higher level of travel risk perception than cluster three. Cluster 3 “The Cautious,” represents 22% of the sample. It showed a relatively high level of perception of COVID-19, whereas the lowest mean of travel risk perception than other clusters. The characteristics of each cluster are shown in Table 2.

**Table 2.** Summary of Cluster Analysis.

Dimension	Cluster 1	Cluster 2	Cluster 3	Total	*F*
	The Anxious	The Reserved	The Cautious		
	*n* = 386	*n* = 227	*n* = 173	*n* = 786	
Perception of COVID-19	4.37	2.58	3.82	3.73	702.439***
Travel risk perception	4.07	3.17	2.60	3.49	386.586***

*** *p* < .001. Wilks’ Lamda = 0.208, *F* = 465.538, *df* = 4 (*p* = .000).

#### Satisfaction Attribute of Destination Image

The exploratory factor analysis was performed to create correlated variable composites from the 17 destination image attributes to generate underlying dimensions. The results showed that eigenvalue exceeding 1 and factor loadings exceeding 0.5. The test value of KMO was 0.926, and the *p*-value of Bartlett’s sphericity test was 6,168.962. The cumulative variance explained is 62.014%. Reliability analysis was also conducted to test the internal consistency retained in each dimension, as shown in Table 3.

**Table 3.** Descriptive of Major Dimensions With Corresponding Reliabilities.

Dimension	Measurement Items	*M*	*SD*	Cronbach’s α
Social environment	Good quality restaurant	4.211	0.145	.846
	Landmark architectures			
	Nice nightlife			
	Interesting cultural events/activities			
	Beautiful scenery/unique natural beauties			
	Various outdoor activities			
Tourist environment	Good local transportation network	4.499	0.063	.775
	Quality infrastructure			
	Convenient public transportation			
	Safe infrastructure			
Destination environment	Enough guides/tours	3.765	0.205	.727
	Good network of tourist information			
	Reasonable price			
Imagery	Pleasant	4.010	0.281	.833
	Exciting			
	Arousing			
	Relaxing			

### A Comparative Analysis of Destination Image Based on Revised IPA

The data were analyzed using Statistical Products and Services Solutions (SPSS). First, the mean and standard deviation of 17 satisfaction attributes related to the destination image of Korea were calculated. Second, the two-staged method proposed by Deng (2007) was performed to measure the implicitly derived importance from the performance items. According to the difference in risk perception in COVID-19 tourism, the performance and implicitly derived importance with destination image satisfaction attributes were produced. The mean scores for all 17 destination image attributes ranged from a high of 4.60 to a low of 3.55. Overall satisfaction’s mean and standard deviation were 4.35 and 0.654, respectively (see Table 4). Cluster 1 “The Anxious,” showed that the attributes “Quality infrastructure,”“Good local transportation network,”“Convenient public transportation,”“Beautiful scenery/unique natural beauties,”“Safe infrastructure,” and “Landmark architectures,” were above the overall satisfaction mean scores. However, the attributes ranked under six had lower mean scores than the overall mean scores. The most important attribute was shown to be “Safe infrastructure” (*M* = 0.172), and the least important attribute was “Good local transportation network” (*M* = -0.091). The results of cluster 2 “The Reserved,” indicated that the attributes “Good local transportation network,”“Safe infrastructure,”“Quality infrastructure,” and “Convenient public transportation” had higher overall satisfaction mean scores. The scores of implicit importance ranged from −0.125 (Safe infrastructure) to 0.195 (Good network of tourist information). Cluster 3 “The Cautious,” revealed that the significant overall satisfaction mean scores of attributes were listed in order of significance: “Quality infrastructure,”“Good local transportation network,”“Safe infrastructure,”“Beautiful scenery/unique natural beauties,” and “Convenient public transportation.” The most significant attribute was shown to be “Reasonable price” (*M* = 0.170), and the least significant attribute was “Beautiful scenery/unique natural beauties” (*M* = −0.130).

**Table 4.** The Performance and Implicitly Derived Importance with Destination Image Attributes.

Items (*n* = 786)	Cluster 1: The Anxious (*n* = 386)					Cluster 2: The Reserved (*n* = 227)					Cluster 3: The Cautious (*n* = 173)				
	Satisfaction performance			The implicitly derived importance		Satisfaction performance			The implicitly derived importance		Satisfaction performance			The implicitly derived importance	
	*M*	*SD*	Rank	Mean (partial correlation)	Rank	*M*	*SD*	Rank	Mean (partial correlation)	Rank	*M*	*SD*	Rank	Mean (partial correlation)	Rank
Good quality restaurant	4.34	0.708	7	.138	3	4.26	0.759	7	.065	6	4.21	0.687	9	.071	7
Landmark architectures	4.37	0.707	6	.070	7	4.23	0.793	9	.015	10	4.25	0.666	7	−.034	13
Nice nightlife	4.10	0.894	11	−.020	15	4.01	0.895	11	.030	9	3.99	0.876	13	.019	10
Interesting cultural events/activities	4.27	0.797	8	.014	13	4.09	0.905	10	.034	8	4.14	0.845	11	−.115	16
Beautiful scenery/unique natural beauties	4.43	0.708	4	.013	14	4.33	0.735	5	.042	7	4.40	0.705	4	−.130	17
Various outdoor activities	4.07	0.932	12	.059	8	4.01	0.917	12	−.029	13	4.01	0.883	12	.121	3
Good local transportation network	4.58	0.684	2	−.091	17	4.50	0.778	1	−.026	12	4.57	0.658	2	−.053	15
Enough guides/tours	3.75	0.929	15	.017	12	3.67	0.968	16	−.042	15	3.94	0.833	14	.073	6
Quality infrastructure	4.60	0.618	1	−.022	16	4.46	0.794	3	−.059	16	4.58	0.639	1	.009	12
Good network of tourist information	3.95	0.990	13	.080	6	3.83	0.986	14	.195	1	4.17	0.824	10	.012	11
Reasonable price	3.54	0.993	17	.059	9	3.51	1.015	17	−.029	14	3.65	0.986	17	.170	1
Convenient public transportation	4.46	0.713	3	.082	5	4.42	0.768	4	.072	5	4.36	0.784	5	.044	8
Safe infrastructure	4.42	0.799	5	.172	1	4.50	0.743	2	−.125	17	4.50	0.653	3	.090	5
Pleasant	4.23	0.714	9	.135	4	4.29	0.730	6	.135	2	4.27	0.691	6	.091	4
Exciting	4.20	0.760	10	.051	10	4.25	0.776	8	.129	3	4.24	0.723	8	.156	2
Arousing	3.93	0.880	14	.170	2	3.86	0.930	13	−.003	11	3.85	0.793	15	−.048	14
Relaxing	3.60	1.152	16	.019	11	3.70	1.125	15	.120	4	3.80	0.976	16	.035	9

*Note*. Destination image attributes were scored on a 5-point Likert scale ranging from (1) not at all satisfied to (5) extremely satisfied. Overall performance satisfaction mean = 4.35.

### Comparative Analysis of Destination Image Based on COVID-19 Tourism Risk Perception

**Figure 1.**
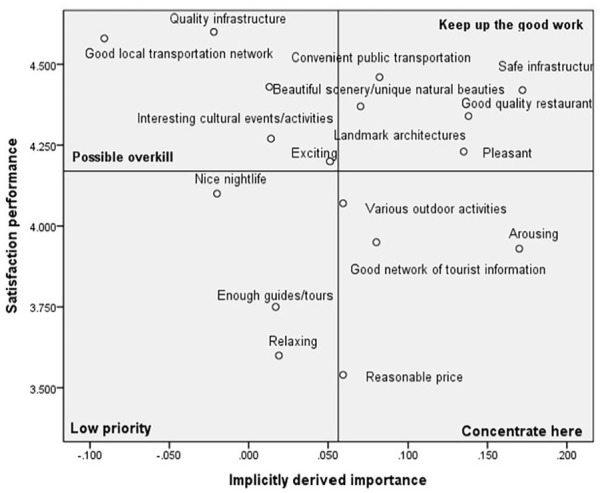
Revised IPA grid for Cluster 1: The anxious.

**Figure 2.**
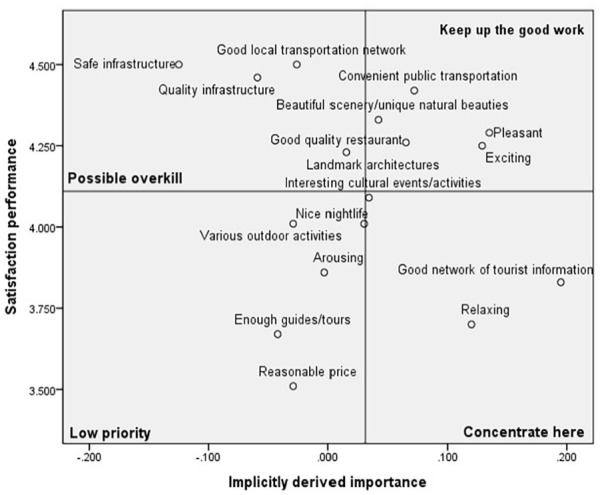
Revised IPA grid for Cluster 2: The reserved.

**Figure 3.**
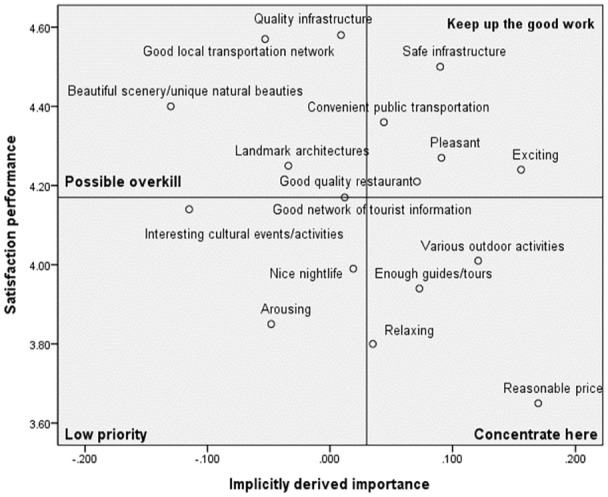
Revised IPA grid for Cluster 3: The cautious.

The 17 destination image satisfaction attributes were identified on the revised IPA grid after obtaining all destination image attributes’ implicitly derived importance and satisfaction performance. The grand means for implicitly derived importance and satisfaction performance are used to place the axes on the grid. The two-dimensional grid showed the satisfaction performance on the vertical axis from extremely high to low (top to bottom) and implicitly derived importance on the horizontal axis from extremely high to extremely low (right to the left). According to the perception of COVID-19 and travel risk, IPA girds for each cluster are presented in Figures 1 to 3.

#### Cluster 1: The Anxious

Figure 1 shows that all attributes were identified in each quadrant. The performance of the items in the Keep up the good work and low priority quadrants appeared to bring balance to what the international students considered important. The attributes plotted in the Possible overkill quadrant were delivered much more than their importance warranted. This result indicated that these attributes were not required more attention and resources to satisfy international students. The attributes in the Concentrate here quadrant were rated above average regarding implicitly derived importance but below average for satisfaction performance. The result indicates that international students perceived these attributes as important but did not consider them executed satisfactorily, given their level of importance. Arousing is the most crucial attribute for international students, and it is suggested that improvement of efforts and special attention should be given to focus more attention on tourism program content and operations for international students. Since Good network of tourist information and Various outdoor activities are also important attributes, it is suggested that tourism practitioners should develop various outdoor activities/programs and provide helpful tourism information for international students. Regarding Reasonable price, the last important attribute, business managers, or tourism practitioners should adjust prices according to the international students’ perceptions of value.

#### Cluster 2: The Reserved

The fiver attributes located in the Keep up the good work quadrant are recognized to be important to international students, and at the same time, they are relatively satisfied with these attributes. The Low priority quadrant captured five attributes that are not necessarily important for international students who are not particularly satisfied with the attributes. The attributes shown in the Possible overkill quadrant indicated that they did not need more attention to satisfy the tourists as these attributes are delivered high levels of satisfaction. In the Concentrate here quadrant, Good network of tourist information is the most important attribute, and it is suggested that the improvement of efforts should be given to developing tourism information systems for international students, and pay attention to consider how to enable international students to contact and reach tourism information and service in Korea easily. The following important attributes are Relaxing and Interesting cultural events/activities. Thus, special attention should be provided to develop tourism programs/content of cultural events/activities to make international students feel relaxed and interested (see Figure 2).

#### Cluster 3: The Cautious

Figure 3 showed that the attributes presented in the Keep up the good work and Low priority quadrant appeared to be considered the needs of international students and balanced to maintain the service level. In the quadrant of Possible overkill, the attributes have high levels of performance and satisfaction despite slight importance to international students. The attributes captured in Concentrate here are important attributes for international students and significant indicators to improve the competitiveness of destination image in Korea.

## Discussion and Conclusion

The purpose of this study was to evaluate the importance-performance ratings of Korea’s destination image attributes in order to determine which elements were seen as very successful, low priority, over-allocated, or improvement required. IPA is one of the most acceptable procedures because of its ease of use and a high diagnostic value (Martilla & James, 1977). However, without a segmentation component, IPA has little practical utility (Bruyere et al., 2002), and combining various segments might yield deceptive results (Farnum & Hall, 2007). Researchers can use market segmentation to analyze the importance and performance diversity of consumers and readily grasp sample characteristics. (Caber et al., 2012). In this regard, this study employed revised IPA and examined segmented groups one by one. The analytical findings revealed that the importance-performance ratings of the attributes for the market segmentation were different. In line with the previous findings, the results of this study showed that the perception of risk is linked to the image of a destination (Carballo et al., 2022; Zhang et al., 2019), and the impact of the perceived risk on the image affects the level of satisfaction (Chi & Qu, 2008).

Cluster 1 “The Anxious” is the first group with a high-risk perception for both COVID-19 and traveling. Arousing, an affective attribute, came out as the most complementary attribute among the attributes. The finding aligns with the previous studies that the affective image had more significant effects than the cognitive image on behavior intentions (Afshardoost & Eshaghi, 2020; Hudson et al., 2015). Cluster 2 The Reserved showed the lowest risk perception for COVID-19 and considered travel risk perception relatively higher than COVID-19 risk perception. In order to increase customer satisfaction in COVID-19 tourism for those who perceive a higher risk perception of traveling, good network of tourist information that allows safe travel should be provided and encourage them to participate in various cultural events and activities at tourist destinations. Moreover, for those with a high travel risk perception that needs to be relaxed on travel, various tourism programs/content should be developed according to their needs. Regarding cluster 3 “The Cautious,” the risk perception for COVID-19 is relatively high, whereas the travel risk perception is the lowest value. This group tends to pursue satisfaction through traveling despite recognizing the COVID-19 risk. Among the attributes of Concentrate here, the most complementary attribute is Reasonable price. As international students serve many roles at a host destination for a longer time, such as students and tourists, they might minimize expenditures on commercial loadings or heavy tourist shopping (Song & Bae, 2018). Money might negatively influence the destination image since money is a critical attribute that influences travel decision-making (S. Huang & Hsu, 2009) and is an important behavioral inhibitor (Atadil et al., 2017).

Each cluster’s common attributes and differences at the dimension level are presented in Table 5. The common attributes of the destination image indicated that the international students were satisfied with Good quality restaurant, whereas they were not interested in Nice nightlife. This finding shows that the destination was recognized as a typical vacation spot preferred by customers seeking relaxation, and the primary motivation for their trip might have been to relax rather than recreation (Caber et al., 2012). In addition, the Korean government implemented strict social distancing rules across the nation, such as business hour curfews and the limitation of private social gatherings (Lim et al., 2021), and these COVID-19 mandated social restrictions might cause to not being interested in Nice nightlife in COVID-19 tourism. In addition, the international students were satisfied with Convenient public transportation, yet they were not interested in Good local transportation network and Quality infrastructure. This finding supports the previous research findings that tourists are unconcerned about transit infrastructure and quality (Nikjoo & Ketabi, 2015). The infrastructure aspect of the cognitive destination image does not significantly affect recommendations and revisit (Akgün et al., 2020). The attribute of Pleasant is commonly located in Keep up the good work, as international students feel pleasant with the destination image of Korea. In this regard, shaping an affective image should be considered a critical attribute because feelings rather than beliefs or actions more directly predict tourists’ decision-making,

**Table 5.** Differences of Three Clusters According to COVID-19 Risk Perception.

Dimension	Measurement items	Cluster 1	Cluster 2	Cluster 3
		The Anxious	The Reserved	The Cautious
Social environment	Good quality restaurant	KG	KG	KG
	Landmark architectures	KG	PO	PO
	Nice nightlife	LP	LP	LP
	Interesting cultural events/activities	PO	CH	LP
	Beautiful scenery/unique natural beauties	PO	KG	PO
	Various outdoor activities	CH	LP	CH
Tourist environment	Good local transportation network	PO	PO	PO
	Quality infrastructure	PO	PO	PO
	Convenient public transportation	KG	KG	KG
	Safe infrastructure	KG	PO	KG
Destination environment	Enough guides/tours	LP	LP	CH
	Good network of tourist information	CH	CH	LP-PO^a^
	Reasonable price	CH	LP	CH
Imagery	Pleasant	KG	KG	KG
	Exciting	PO	KG	KG
	Arousing	CH	LP	LP
	Relaxing	LP	CH	CH

*Note*. LP = low priority; PO = possible overkill; KG = keep up the good work; CH = concentrate here.

a Borderline between two dimensions.

## Theoretical and Managerial Implications

The objective of this study was to compare differences in destination image assessment for international students based on their perception of COVID-19 and travel risk. The distinctive clusters provided valuable insights and contributed to a better understanding of the relationship between risk perception and destination image. This study produced a number of theoretical contributions to the academic literature of health crises, travel risk perception, and destination image by classifying international students based on their risk perception in COVID-19 tourism and its impact on the assessment of destination image. The cluster analysis discovered different clusters, and the comparison of results showed that satisfaction with the destination image appears differently according to the degree of COVID-19 risk perception and travel risk. From a practical perspective, the results of this study provide some managerial insights to destination marketers and tourism organizations to develop communication strategies for the second wave of the COVID-19 crisis, which health authorities have highlighted (Bae & Chang, 2021). First, tourism practitioners should focus on minimizing travel risk perception to have the tourism business recover swiftly once the threat of COVID-19 has decreased. Second, tourism organizations may need to prepare for a new paradigm that accommodates tourists’ needs to satisfy their travel. In addition, communication strategies should focus on information on tourism policies about cancellation or refunds as well as health and safety requirements. Third, destination branding marketers need to consider international students as long-term tourists whose information about the destination is reliable and concentrate on revitalizing domestic tourism and realigning host destinations with the changing international tourism industry.

Despite the theoretical and practical implications, there are several limitations. First, this study employed convenience sampling, which lessens the generalizability of the findings to a broader sample due to the uneasiness of sampling. The perceived importance and satisfaction may differ with a larger sample of international groups such as expatriates, immigrant workers, or multicultural families during the research design and sampling process. Second, The sample is confined to the impressions of international students at a single moment in time during the epidemic and is not entirely representative of the worldwide population of prospective tourists. Future studies will likely put longitudinal studies or data collecting at numerous stages during the COVID-19 epidemic to the test. In addition, future studies would need to consider more demographic characteristics of respondents to make comparisons in destination marketing. It would expand understanding of international students’ perception of destination image and develop strategies targeting the market for tourism organizations and local governments.
